# Establishing Norms of Connected Speech Measures for Story‐Telling in Cantonese‐Speaking Adults

**DOI:** 10.1111/1460-6984.70131

**Published:** 2025-09-30

**Authors:** Anthony Pak‐Hin Kong, Cherie Wan‐Yin Wong, Chester Yee‐Nok Cheung

**Affiliations:** ^1^ Academic Unit of Human Communication, Learning, and Development (HCLD), Faculty of Education The University of Hong Kong Pokfulam Hong Kong; ^2^ The Aphasia Research and Therapy (ART) Laboratory The University of Hong Kong Pokfulam Hong Kong

**Keywords:** ageing, Cantonese, narrative discourse, norms, story‐telling

## Abstract

**Background and Objectives:**

Narrative discourse is a useful means to organize ideas and create shared understandings. Clinically, performing discourse analysis on disordered spoken language could facilitate researchers and clinicians not only to evaluate one's language abilities but also to foreshadow his/her communication in real‐life situations. Given the normative reference data of a specific discourse task, less‐biased judgement and evaluation could be made, which could further facilitate assessment and intervention planning. This study aims to first develop norms by analysing the language samples produced by neurotypical Cantonese speakers on two well‐familiarized narrative stories, The Boy Who Cried Wolf, and The Tortoise and Hare. Second, we aim to investigate the potential age and education effects on a wide range of micro‐ and macro‐structural linguistics measures.

**Method:**

Two semi‐spontaneous story narratives from the Cantonese AphasiaBank were selected for scoring. A total of 150 neurotypical Cantonese adult speakers produced the spoken discourse samples for each story narrative. All speakers were native Cantonese speakers living in Hong Kong; they were divided into three age groups: young (18–39 years old), middle‐aged (40–59 years old), and older (> 60 years old). Audio recordings were transcribed, segmented, and annotated using CHAT conventions.

**Results:**

Normative references of various micro‐ and macro‐structural linguistics measures and the standard scoring references for the two narrative stories were established. For the age effect on narrative discourse, the older adults produced less complex, coherent and thematic‐related concepts compared to the young group. However, lexical diversity was preserved in the older group, resulting in no significant differences across the three age groups. For education effect, the higher education group outperformed the lower education group in verbal productiveness and content informativeness. Lastly, the two stories were found to be non‐comparable to each other, thereby they should not interchange in pre‐ and post‐test arrangements or in monitoring discourse performance.

**Conclusions:**

The Cantonese discourse norms presented here can be applied in both research and clinical settings, facilitating a more objective review of language impairment and treatment planning. Second, this study demonstrated the effect of normal ageing on both the linguistics and conceptual levels specific to discourse production.

**WHAT THIS PAPER ADDS:**

*What is already known on this subject*

- Discourse analysis is a critical part of evaluating and understanding a person's communication abilities. Studies indicated that narrative discourse is more sensitive to specific linguistic parameters than other genres, and people with stronger narrative skills tended to have more social communication opportunities. An increasing number of studies had been working on setting norms for discourse tasks. Locally, Kong et al. (2025) have recently reported the normative references for descriptive tasks of the Cantonese AphasiaBank.

*What this study adds to the existing knowledge*

- First, this study completed the norms establishment for all discourse tasks of the Cantonese AphasiaBank. Second, our analysis of the impact of different factors on narrative discourse offers significant value for clinical applications. We found that ageing was not manifested across all microstructural linguistics consistently, while lexical diversity was found to be tolerant to ageing. However, ageing was found to be adversely affecting propositional parameters and discourse informativeness.

*What are the clinical implications of this study?*

- Narrative discourse, being one of the most popular tasks in clinical assessment but often faced the challenges of a lack of objective references. This study analysed two well‐familiarized narrative stories, provides a complete set of norm data readily for front line clinicians and researchers, which could be used for intervention planning, monitoring progress (e.g., used them as control probes for tracking generalization effects) or as an input for investigating the interplay between linguistics and cognitive abilities.

## 1 Introduction

Discourse is a language unit beyond a single utterance or sentence (Kong 2022). It includes diversified communication acts such as conveying and receiving information in daily social interactions.

McCabe and Bliss (2006) identified four genres of discourse: (i) conversation, (ii) script, (iii) narrative, and (iv) procedural discourse. Studies suggest that different discourse genres can influence the range of spoken output (Kong et al. 2025; Pritchard et al. 2017; Olness 2006), with each genre offering unique advantages. For example, conversational discourse elicits less constrained productions, reflecting a more realistic communication performance in everyday situations (Cruice et al. 2000). In contrast, genres such as picture description, procedural discourse, and narrative re/telling result in more structured outputs, providing a common reference point that facilitates comparisons of performance over time and across different groups (Bryant et al. 2016; Kong 2022). In light of the uniqueness of different genres, analysing discourse provides a wide range of information about a person's linguistics, functional and pragmatic communication abilities.

Aphasia is an acquired language disorder that adversely impairs one's receptive and expressive abilities, ranging from word level to sentence level to discourse level (Kong 2022). Compared to neurologically intact adults, people with aphasia (PWA) often experience difficulties in discourse productions, types of words, lexical diversity, informativeness and correctness and so forth, which are greatly reduced with increasing aphasia severity (e.g., Fergadiotis and Wright 2011; Nicholas and Brookshire 1995). Given that discourse production is closely linked to everyday life, PWA often reflect lower social participation and activities (Dalemans et al. 2010), communication confidence and quality of life (Yaqoob et al. 2021).

### 1.1 Discourse Analysis

Discourse analysis has gained increasing importance in studies related to cognition (Dutta et al. 2024), the geriatric population (Wright et al. 2005), and aphasia (Bryant et al. 2016). In both clinical practice and research of aphasia, discourse analysis serves as an essential methodology for evaluating language abilities, addressing the limitations associated with using discrete language tasks, which are frequently intended to measure proficiency in particular language components rather than capturing the more complex dynamics of language use (Bryant et al. 2016). Though Herbert et al. (2008) observed a significant correlation between picture‐naming abilities and some discourse measures in conversation tasks, a former study conducted by Mayer and Murray (2003) suggested an insignificant correlation between confrontation naming and connected spoken language samples. The discrepancy of results might be subjectively attributed to methodological issues, such as the choice of discourse measures and tasks in studies. This leads to the need of cautious selection of discourse tasks in assessment and adapting a comprehension array of measures for analysis. From a practical perspective, some discourse measures, like main concept analysis (MCA) (Ross and Wertz 1999) and linguistic indices (Kong and Wong 2018), were found to be significantly correlated to naïve listener ratings, suggesting that discourse analysis might better predict changes in real‐life communication exchange than standardized tests presenting an overall score (Ross and Wertz 1999).

Most widely used standardized assessment tools in aphasia, such as Western Aphasia Battery‐Revised (WAB‐R; Kertesz, 2007), Comprehensive Aphasia Test (CAT; Swinburn et al. 2004), commonly include a picture description task to elicit discourse production. However, these assessments often rely on subjective rating, which hinders tracking performance changes and maintaining a consistent and objective evaluation. Moreover, the lack of normative references for these discourse tasks complicates understanding how and to what extent pathological spoken language sample deviates from normal performance. Given this, several research studies have been conducted to establish normative references for discourse tasks; examples included the Cookie Theft task of Boston Diagnostic Aphasia Examination in English (Berube et al. 2019) and in French‐Canadian population (Boucher et al. 2022), discourse tasks from AphasiaBank database (http://talkbank.org/AphasiaBank/) (Richardson and Dalton 2016), and so forth. Kong et al. (2025) investigated and established normative references for five discourse tasks (i.e., two single picture description tasks, two sequential picture description tasks, and one procedural description task) from Cantonese AphasiaBank (Kong and Law 2019), depicting the discourse abilities of healthy adults in respect to three age subgroups, education level, and genre differences. This study analyses the narrative discourse abilities of Cantonese‐speaking healthy adults, which has not been covered in the study of Kong et al. (2025), to expand the bank of normative data in Chinese aphasiology.

### 1.2 Narrative Discourse and Its Clinical Applications

Narrative discourse, which encompasses storytelling and personal narration, involves presenting events or experiences in a time‐sequenced manner (Spencer and Slocum 2010). Beyond the purpose of completing the establishment of language‐specific normative references for various discourse genres, narrative discourse offers significant value for clinical applications. First, narrative discourse is more sensitive to specific linguistic parameters than other genres. Specifically, Pritchard et al. (2017) observed that narrative discourse provided richer semantic frameworks than procedural description. Similarly, Ulatowska et al. (1983) showed that narrative discourse often exhibited greater syntactic complexity compared to procedural discourse. Second, narrative discourse analysis provides additional information related to one's cognitive abilities and social participation, which could be factors to be concerned about in treatment planning. Research by Hilviu et al. (2024) explored the relation between age, narrative spoken discourse and cognitive functions. Though the study did not compare narrative discourse with other genres, it suggested the strong correlation between narrative discourse, especially the macrostructural linguistics measures with theory of mind, and executive functions (such as long‐term memory and inhibitory control). Regarding participation level, Armes et al. (2020) investigated the relationship between macrostructural linguistics variables (i.e., MCA variables) of narrative discourse and some quality‐of‐life measures, and the results suggested a moderate correlation between the main concept (MC) composites and assessment for living with aphasia, communication confidence and community integration.

### 1.3 Narrative Discourse, Age, and Education

Narrative production ability in normal ageing has been extensively documented, particularly in English‐speaking populations. Various narrative tasks have been employed, such as storytelling from wordless picture books, videos, and cartoons or retelling familiar stories (e.g., the Cinderella story). Research has examined a wide range of story production measures in the context of ageing. For example, lexical diversity (Fergadiotis et al. 2011; Kemper and Sumner 2001) and semantic content (Juncos‐Rabadán et al. 2005) are unaffected by healthy ageing. In contrast, syntactic complexity (Hilviu et al. 2024), the ability to produce correct informative units, content accuracy, and completeness tended to decline with age (e.g., Capilouto et al. 2005; Wright et al. 2005). These findings suggested that not all narrative skills are adversely affected by ageing, leading to the need for comprehensive analysis to understand what is being affected and how.

Research on narrative abilities and ageing often not only examine one's language abilities but also investigate discourse abilities in relation to cognitive abilities. Kemper et al. (1990) found that older adults showed reduced syntactic complexity and cohesive ties when retelling a story from a cartoon, with working memory identified as a contributing factor. Wright et al. (2011) compared story production abilities between young adults (ages 20–29) and older adults (ages 70–89) using wordless picture books, and the results revealed significant differences in story measures caused by memory and attention span discrepancy. Hilviu et al. (2024) assessed storytelling abilities across three age groups: 20–40, 65–75, and 76–86 years old, using specific prompts. The study evaluated the interplay between micro‐ (i.e., lexical and grammatical) and macro‐structural (i.e., pragmatic and discourse) linguistics variables alongside cognitive skills. Findings of that study indicated diminished performance of syntactic complexity, coherence, and content informativeness in the older adult group and highlighted a strong correlation between narrative skills and theory of mind. Though these studies did not compare narrative discourse with other discourse genres, they emphasize the importance of considering age‐related cognitive changes when analysing narrative productions, which is especially important when evaluating pathological spoken language samples for determining the severity of impairment.

Studies have highlighted the significant impact of education on narrative production abilities. A recent systematic review conducted by Malcorra et al. (2022) analysed the influence of education and literacy in older adult narrative abilities, and the results showed that individuals with higher education levels tended to excel more than the lower education groups in measures such as informativeness and story structure. This pattern aligned with earlier research studies, including that by Juncos‐Rabadán (1996), who employed personal storytelling tasks, and the study of Le Dorze and Bédard (1998), who used single‐picture stimuli to elicit narrative productions and found level of education affects the wealth of content conveyed. Pereira and Ortiz (2022) conducted a comprehensive assessment of oral and written language skills to investigate the effects of education level. Specifically looking into the oral narrative task they performed, their findings indicated that adults with lower education performed poorer in oral discourse, such as making fewer propositions and exhibiting lower coherence, suggesting a weaker mental representation made between explicit linguistic information and world knowledge of the group. These findings suggest a complex inter‐relation between education and healthy ageing. Therefore, considering education level as a factor in analyses could help develop more rigorous normative references for narrative abilities.

### 1.4 Multilevel Analysis of Narrative Samples

As summarized by Kong et al. (2025), a comprehensive multilevel (i.e., micro‐ and macro‐structural linguistic variables) analysis of connected speech language samples would facilitate the understanding of how various linguistic or propositional variables were affected (or not affected) throughout the life span. This approach guides future discourse research, particularly in evaluations and treatment planning. For pathological spoken language samples, Wirz (1995) suggested that such multilevel discourse analysis enabled differential communication disorder diagnosis, even among individuals with similar discourse breakdown. More recent studies support this notion. Kong (2011) demonstrated that micro‐ and macro‐structural linguistic features (e.g., lexical richness, number of accurate and complete concepts) could effectively assess language performance, even in individuals with the mildest forms of aphasia.

Additionally, macrostructural linguistic parameters, such as information content words and MCs, have been shown to capture changes in oral discourse associated with Alzheimer's disease (Ahmed et al. 2013) and correlate significantly with aphasia severity (Fromm et al. 2017; Kong and Law 2009; Kong and Wong 2018). Furthermore, recent research conducted by Kim et al. (2019) applied multilevel analysis on three discourse tasks for differential diagnosis of healthy ageing, amnestic and non‐amnestic mild cognitive impairment (MCI). They identified some measures (e.g., cohesive words and propositions were worse in amnestic than non‐amnestic MCI) that were more prominent in distinguishing between these groups. Overall, these studies illustrated that discourse measures have different effects in differentiating between normal‐ageing and disordered spoken language samples. Therefore, a comprehensive, multilevel analysis is essential for establishing normative references in discourse production.

### 1.5 Aims

1. To extend the establishment of normative reference to narrative discourse for the Cantonese‐speaking population in Hong Kong, Kong et al. (2025) presented a comprehensive quantitative discourse analysis for picture descriptions and procedural discourse, clearly depicting the linguistics and proposition features. This study targets expanding the bank of normative data in Chinese aphasiology in a consistent and congruent manner.
2. To investigate how age and education affect one's performance in narrative discourse. Previous studies noted age and education effects (e.g., Kong et al. 2025; Pereira and Ortiz 2022). This study also intends to see how age and education affect narrative discourse's micro‐ and macro‐structural linguistics variables.

## 2 Method

This study aims to perform a multilevel (i.e., to operationalize micro‐ and macro‐structural linguistics variables) discourse analysis of two classic stories: ‘The Boy Who Cried Wolf’ and ‘The Tortoise and Hare,’ utilizing the 150 spoken language samples extracted from Cantonese AphasiaBank (www.speech.hku.hk/caphbank/search/; Kong and Law 2019).

### 2.1 Participants

One hundred fifty native Cantonese‐speaking neurotypical adults participated in a series of discourse productions for different genres, and only the narrative stories were extracted for this study. For detailed inclusion and exclusion criteria, please refer to Kong et al. (2025).

Participants were divided into three age groups: young (aged 18 to 39, *N* = 48, 24 women, *M*_age_ = 26.50 ± 6.38 years, *M*_edu_ = 13.38 ± 2.74 years), middle‐aged (aged 40 to 59, *N* = 48, 24 women, *M*_age_ = 49.85 ± 4.88 years, *M*_edu_ = 12.38 ± 3.27 years), and old (60 or above, *N* = 54, 28 women, *M*_age_ = 66.07 ± 5.22 years, *M*_edu_ = 8.46 ± 3.94 years), and two education levels: low (*N* = 79) and high (*N* = 71). Based on Hong Kong's literacy rate, a high education level was defined as higher than secondary school or having received at least 14 years of education for the young and middle‐aged groups and higher than primary school or at least 7 years of education for the old group (Kong and Law 2019). The details of participants' demographic information are shown in Table 1.

**TABLE 1:** Distribution of participants by age, gender, and education.

Age group	Male		Female		
	Low education	High education	Low education	High education	Subtotal
Young (18–39 years old)	12	12	12	12	48
Middle‐aged (40–59 years old)	16	8	15	9	48
Old (60 years old or above)	11	15	13	15	54
Subtotal	39	35	40	36	**Total = 150**

*Note*: In defining ‘Low’ and ‘High’ education, this study aligned with the study of Kong and Law (2009), in which 14 years of education is considered as high for the two younger groups, while 7 years of education is deemed as high for the old group. According to the population census released by the Hong Kong Special Administrative Region (2022), more than 70% of the population in the 1970s only received 6 years or lower education while about 50% of the population received 13 years of education in the 2020s. Therefore, the high illiteracy among native Cantonese elderly speakers limited the applicability of a uniform high‐low education criterion in the old group. Furthermore, many job positions that previously required secondary education now required tertiary education. We posit with the belief mentioned in Kong and Law (2009) that language abilities of our old participants are linked with education level and working experiences.

### 2.2 Data Collection

All participants in the study had signed consent and agreed to have their spoken language productions video recorded. The data collection and transcription procedures followed the Cantonese AphasiaBank protocol (Kong and Law 2019). All participants were presented with the picture books of the two stories. No time constraint had been imposed on how long they could read and organize their spoken language. The picture book was removed once the participant was ready to tell the story. Each spoken language sample was de‐identified and coded to ensure confidentiality. All personal demographic information, as well as the dates and times of the recording, was encrypted and accessible only to the primary and secondary invigilators and authorized researchers. A more detailed transcription procedure could refer to Kong et al. (2025).

### 2.3 Microstructural Linguistics Variables

Table 2 summarizes the microstructural linguistics variables into four linguistics categories, consistent with Kong et al.'s (2025) approach. The definition of the microstructural linguistics variables used in this study is also explained in Table 2. All these variables were calculated using the CLAN software (MacWhinney 2000).

**TABLE 2:** Quantitative linguistic analysis: Linguistics categories and their measures and definitions.

		Micro‐structural linguistics variables	Definition
Linguistics categories	Productivity	Duration	The total time taken for the spoken narrative, measured in second.
		Total utterances	An utterance is counted as a verb or a clause per line (Kong and Law 2009 ). For example: 不如_1_ 嗌_2_「 狼_3_ 來_4_ 了_5_」^*^ contains a total number of 1 utterance.
		Mean length utterance‐words	It is calculated by dividing the total number of words by the total number of utterances. For example, 不如_1_ 嗌_2_「 狼_3_ 來_4_ 了_5_」^*^ contains five words, the mean length utterance‐words is 5.
		Types	It refers to the unique forms of words in a spoken language sample. Each unique word is counted only once, regardless of how many times it has been produced in the spoken language sample. For example: 佢_1_ 呢_2_ 就_3_ 為_4_ 咗_5_ 想 [/] 想_6_ 呢_7_ 開_8_ 玩笑_9_ ^**^ contains a total number of 8 types.
		Tokens	It refers to the total number of words being produced in a spoken language sample, including repetition. For example: 佢_1_ 呢_2_ 就_3_ 為_4_ 咗_5_ 想 [/] 想_6_ 呢_7_ 開_8_ 玩笑_9_ ^**^ has a total number of tokens equals to 10.
		Tokens per minute	It evaluates a spoken language sample by calculating the number of words produced in 1 min.
	Syntactic complexity	Verbs per utterance	It measures the number of verb(s) in each utterance. A higher count may indicate a greater syntactic complexity because more verbs may infer more use of compound or complex structures.
		Noun‐to‐verb‐ratio	The ratio compares the number of nouns to the number of verbs in the spoken language sample. A higher ratio usually infers a more descriptive style of spoken language sample, while a lower ratio might be more action‐oriented.
		Open‐to‐closed‐class ratio	The ratio compares the number of open‐class words (e.g., nouns, verbs, adjectives etc.) to closed‐class words (e.g., aspect markers, final particles, conjunctions etc.). A higher ratio indicates greater complexity as open‐class words often lead to the use of more complex phrases, clauses and elaborative constructions.
	Dys‐fluency	Retracing	It refers to the going back of previous point in spoken language sample for clarification or corrections.
		Repetitions	It refers to the repeating of words, phrases or an entire sentence.
	Lexical diversity	Type‐token ratio	It is calculated by dividing the types by the total number of tokens. For example: 佢_1_ 呢_2_ 就_3_ 為_4_ 咗_5_ 想 [/] 想_6_ 呢_7_ 開_8_ 玩笑_9_ ^**^ yields a type‐token ratio of 0.8.
		VocD	The VocD's program in CLAN estimates lexical diversity by taking 100 random samples of 35 tokens and calculating a mean type‐token ratio for the samples; then the procedure repeats for samples of 36 tokens till samples of 50 tokens (McCarthy and Jarvis 2007)

* 1connective|bat1jyu4 = why not; ^2^verb|ngaai3 = shout; ^3^noun|long4 = wolf; ^4^verb|loi4 = come; ^5^sentence final particle|liu5 = already.

** ^1^pronoun|keoi5 = he; ^2^sentence final particle|ne1; ^3^adverb|zau6 = then; ^4^verb|wai4 = cost; ^5^aspect marker|zo2 = perfective; ^6^auxiliary verb|soeng2 = want; ^7^sentence final particle|ne1; ^8^verb|+v|hoi1+; ^9^n|waan4siu3 = joke.

### 2.4 Macrostructural Linguistics Variables

#### 2.4.1 Information Content Units (ICUs)

ICU is defined as ‘the prescribed units of accurate and relevant information conveyed’ (Cooper 1990). Measuring the ICU of a context (i.e., total ICUs) could reflect the degree of informativeness of a connected spoken language sample. The other ICU‐related measures used in analysis included ICU per second, ICU per word, and ICU per utterance, which reflects communication efficiency.

#### 2.4.2 MCs

MC is defined as ‘statements (that) provide an outline of the gist or essential information portrayed in the stimulus pictures…and should contain one and only one main verb’ (Van Dijk 1980, as cited in Kong 2009). MCA (Kong 2009) adopted in this study is a measurement of proposition, that is, focusing on key concepts and their productions in oral discourse. A proposition is a combination of one predicate plus one or more arguments; each has a relationship to its predicate, such as the agent, experiencer, instrument, object, source, or goal (Jensen et al. 2006). The MC‐related measures in this study included total MC scores, which also reflect the informativeness of a spoken discourse sample, and the accurate and complete concepts per minute (i.e., ACs/min), which measure how efficiently a proposition is conveyed in relation to time.

#### 2.4.3 Checklist Set‐Up

A set of checklists has been set up for ‘The Boy Who Cried Wolf’ and ‘The Tortoise and Hare’, respectively, and they represent the standardized reference of the two stories. Each checklist presents the scenarios, the ICUs in terms of five categories (i.e., subjects, places, objects, actions and others) and the MCs. The frequency refers to the percentage occurrence in our spoken language samples (i.e., a total number of 150). The procedure of setting up the checklists in this study was consistent with that of Kong et al. (2025), following the guidelines of Jensen et al. (2006). The procedure is briefly outlined as follows:

1. Propositionalize the transcriptions.
2. Divide the text into different thematic scenarios.
3. Generate a core proposition checklist by selecting the proposition produced by at least 20% of the participants.
4. Generate the standard ICUs checklist: extract the units from core propositions based on the five categories: *subjects, objects, places, actions*, and *others*.
5. Generate the standard MCs checklist: Extract the MCs produced by at least 60% of the participants (Kong and Wong 2018).

Online appendices 1 and 2 contain the checklists for the two stories ‘The Boy Who Cried Wolf’ and ‘The Tortoise and Hare.’ The alternative acceptable lexicons for MCA of the two stories are listed in Online appendices 3 and 4.

### 2.5 Scoring, Inter‐ and Intra‐Rater Reliabilities

Scoring of the spoken language samples began after the two checklists had been set up. For the ICU, each unit's appearance would be given 1 point. For MCs, MCA (Kong 2009) was employed. Each production was evaluated on its accuracy and completeness in conveying the idea, then scored as one of the following four categories: (1) Accurate and Complete (AC) – 3 points, (2) Accurate but Incomplete (AI) – 2 points, (3) Inaccurate (IN) – 1 point, and (4) Absent (AB) – no point.

After calculating the total ICU and MC scores, a series of macrostructural linguistics variables of communication efficiency were derived. These variables included ICUs per second, ICUs per word, ICUs per utterance, and ACs per minute.

Inter‐rater reliability of ICU and MC scoring was performed using the intraclass correlation coefficient (ICC). Five percent of the samples were randomly extracted and re‐scored by the third author.

### 2.6 Data Analysis

SPSS v.25 was used to conduct a statistical analysis. To establish normative references for Cantonese‐speaking Chinese in story narratives, aligning with Kong et al. (2025), 90% winsorization (Dixon 1980) was applied to extreme scores. The normality of variables was then verified by the Kolmogorov–Smirnov test. To analyse how age and education would affect the formation of a story, ANOVA was used for those normally distributed variables, and the Kruskal–Wallis test and Mann–Whitney test were used for those non‐normal variables, followed by post hoc tests to identify group differences. Lastly, the Friedman test was performed to test if the two stories are comparable to each other in all age and education subgroups.

## 3 Result

### 3.1 Normative Data of the Stories

The normative data of micro‐ and macro‐ structural linguistics variables in the story narrative was established, and the winsorized descriptive statistics for ‘The Boy Who Cried Wolf’ (S1) and ‘The Tortoise and Hare’ (S2) were summarized in Tables S1 and S2, respectively. Regarding normality, even a 90% winsorization had been applied to the data; most micro‐ and macro‐structural linguistics variables were not normally distributed. Most microstructural linguistic variables showed positive skewness, suggesting the mean was pulled up by the presence of some longer and more complex spoken language samples. Analysing productivity, ‘The Boy Who Cried Wolf’ had a higher kurtosis than ‘The Tortoise and Hare’ in duration (Kurtosis_S1_ = 1.29; Kurtosis_S2_ = 0.19), total utterances (Kurtosis_S1_ = 2.06; Kurtosis_S2_ = 0.70) and tokens (Kurtosis_S1_ = 3.38; Kurtosis_S2_ = 0.03), indicating the former story was presented with higher variability and greater extreme values. Regarding syntactic complexity and dysfluency, ‘The Tortoise and Hare’ were noted with higher variability in open‐to‐closed class ratio (Skewness_S1_ = 0.56; Kurtosis_S1_ = −0.52; Skewness_S2_ = 1.13; Kurtosis_S2_ = 0.92) and retracing (Skewness_S1_ = 1.12; Kurtosis_S1_ = 0.13; Skewness_S2_ = 1.25; Kurtosis_S2_ = 1.16). Regarding the results of lexical diversity, both stories were comparable, and the range of vocabulary used tended to be consistent across samples. Regarding the macrostructural linguistics variables, both stories' ICUs and MC scores showed negative skewness, suggesting lower score outliners in the dataset that had pulled the mean to the left. At the same time, ‘The Boy Who Cried Wolf’ was observed with a more pronounced negative skew than ‘The Tortoise and Hare’ in both total ICUs and MC scores, suggesting a larger number of lower score outliners.

### 3.2 Standardized ICUs and MCs Checklist and Reliability Measures

As mentioned in the previous section, standardized ICUs and MC checklists were drawn before scoring. The checklists listed all the ICUs and MCs of the two stories in original Chinese and English‐translated versions; details might refer to Online appendices 1 and 2. The alternative acceptable lexical items for MCA were summarized in Online appendices 3 and 4. ICC of ICU and MC scoring were as follows: ‘The Boy Who Cried Wolf’ had values of 0.87 and 0.93; ‘The Tortoise and Hare’ was 0.91 and 0.89, respectively. The ICC for both stories indicated high inter‐rater reliability.

### 3.3 Effect of Age and Education

#### 3.3.1 The Boy Who Cried Wolf

Table 3 summarizes the results of means, standard deviations, the main effect of age and education and relevant post‐hoc tests. To analyse the age and education effect, one‐way ANOVA was performed for normally distributed variables, which were TTR, tokens/min, noun‐verb ratio, ICUs/s, ICUs/utterance and ACs/min. The non‐normally distributed variables were analysed using the Kruskal–Wallis test for age effect and the Mann–Whitney test for education effect. The significant value of age post hoc had been adjusted to *p* < 0.017 after Bonferroni correction.

**TABLE 3:** Means, standard deviations, and group comparisons of the narrative story – ‘The Boy Who Cried Wolf’.

	18–39 years old group		40–59 years old group		> 60 years old group				
Variables	Y‐Low ed. (*N* = 24)	Y‐High ed. (*N* = 24)	M‐Low ed. (*N* = 31)	M‐High ed. (*N* = 17)	O‐Low ed. (*N* = 24)	O‐High ed. (*N* = 30)	Age main effect^a^, ^b^	Edu. main effect^a^, ^b^	Post‐hoc
Microstructural linguistics									
Duration (s)	56.96 (21.91)	68.83 (22.14)	85.52 (40.17)	91.06 (53.68)	53.92 (19.42)	99.83 (54.70)	^b^ 5.056^*^	^b^ 2.698^**^	Y < M^**^; H > L^**^
Total utterance	23.92 (9.55)	34.42 (11.78)	39.61 (22.43)	41.71 (27.95)	23.50 (10.05)	43.90 (27.12)	^b^ 4.282	^b^ 3.209^**^	H > L^**^
MLU‐Words	7.01 (0.98)	7.04 (1.27)	6.98 (0.91)	6.88 (1.78)	6.47 (0.97)	6.53 (0.85)	^b^ 8.088^*^	^b^ 0.589	Y > O^**^; M > O^**^
Types	78.13 (20.09)	100.46 (17.07)	99.55 (34.13)	106.44 (41.41)	70.03 (19.82)	104.56 (40.13)	^b^ 4.765	^b^ 3.916^**^	H > L^**^
TTR	0.49 (0.08)	0.44 (0.07)	0.41 (0.08)	0.43 (0.08)	0.48 (0.08)	0.42 (0.08)	^a^ 4.391^*^	^a^ 1.851	Y > M^**^
Tokens	167.92 (70.70)	236.08 (64.37)	263.14 (127.10)	267.00 (151.28)	155.39 (61.91)	268.78 (142.81)	^b^ 5.849	^b^ 3.286^**^	H > L^**^
Tokens/min	177.63 (25.92)	209.16 (27.61)	186.42 (24.26)	186.88 (30.86)	173.10 (34.33)	175.08 (36.03)	^a^ 5.841^*^	^a^ 2.080^*^	Y > O^**^; H > L^*^
Verbs/utterance	1.52 (0.35)	1.37 (0.27)	1.43 (0.28)	1.39 (0.36)	1.40 (0.27)	1.36 (0.29)	^b^ 0.834	^b^ 1.877	
Noun‐verb ratio	0.69 (0.14)	0.69 (0.11)	0.64 (0.09)	0.68 (0.10)	0.66 (0.13)	0.64 (0.11)	^a^ 2.158	^a^ 0.428	
O‐C ratio	1.25 (0.21)	1.06 (0.16)	0.96 (0.18)	1.10 (0.23)	1.07 (0.19)	1.03 (0.20)	^b^ 15.170^*^	^b^ 0.299	Y > M^**^; Y > O^**^
Retracing	0.88 (0.90)	0.83 (1.05)	1.59 (1.64)	1.38 (1.51)	0.89 (1.28)	1.53 (1.62)	^b^ 3.156	^b^ 0.240	
Repetitions	0.92 (1.10)	1.39 (1.76)	2.08 (2.30)	2.58 (2.77)	2.31 (2.16)	2.66 (2.40)	^b^ 9.690^*^	^b^ 0.914	Y < O^**^
VocD	49.44 (11.74)	53.05 (11.00)	47.82 (10.88)	51.90 (12.38)	42.70 (12.55)	50.85 (14.37)	^b^ 4.042	^b^ 2.411^**^	H > L^**^
Macrostructural linguistics									
Total ICUs	26.33 (5.35)	30.50 (4.02)	30.33 (6.71)	30.29 (5.34)	21.84 (6.70)	28.12 (6.48)	^b^ 14.476^*^	^b^ 2.507^**^	Y > O^**^;M > O^**^;H > L^**^
ICUs/sec	0.51 (0.16)	0.48 (0.16)	0.41 (0.16)	0.43 (0.20)	0.42 (0.15)	0.39 (0.21)	^a^ 5.544^*^	^a^ 0.542	Y > O^**^
ICUs/word	0.17 (0.05)	0.13 (0.03)	0.13 (0.05)	0.13 (0.06)	0.15 (0.05)	0.13 (0.06)	^b^ 3.998	^b^ 1.811	
ICUs/utterance	1.20 (0.36)	0.97 (0.27)	0.95 (0.40)	0.96 (0.45)	0.97 (0.30)	0.87 (0.43)	^a^ 2.835	^a^ 1.569	
MC scores	27.67 (5.02)	30.00 (3.39)	27.87 (5.22)	28.82 (3.41)	23.71 (7.21)	27.50 (5.53)	^b^ 5.996^*^	^b^ 2.101^*^	Y > O^**^; H > L^*^
ACs/min	10.07 (3.72)	9.03 (2.67)	7.27 (3.23)	8.11 (4.18)	8.33 (3.43)	7.26 (4.34)	^a^ 5.696^*^	^a^ 0.804	Y > O^**^

*Note*: The values are listed in the order ‘mean (standard deviation)’.

Abbreviations: AC = accurate‐and‐complete main concept; ICU = information content unit; M = middle‐aged; MC = main concept; MLU‐Words = mean length utterance (words); O = old‐aged; O‐C ratio = open‐to‐closed class ratio; TTR = type‐token ratio; VocD = lexical diversity; Y = young‐aged.

^a^ = parametric test (i.e., ANOVA) was performed. Age main effect: *F*(2, 147); Education main effect: *F*(1, 148).

^b^ = non‐parametric tests (i.e., Kruskal–Wallis *H* test and Mann–Whitney *U* test) were performed.

* = *p* < 0.05,

** = *p* < 0.017.

Significant age effects were observed in duration, MLU‐words, TTR and tokens/min. Post hoc analyses revealed that the middle‐aged group had significantly longer duration time than the young group. Both the young and middle‐aged groups significantly produced more MLU words than the old‐aged group. The young group showed higher TTR and tokens/min than the middle‐aged and old‐aged groups, respectively.

Significant age effects were also found in the open‐to‐closed class ratio and repetition. Post‐hoc analyses of the former measure suggested that the young group utilized more open‐class words (or fewer closed‐class words) than the middle‐aged and old‐aged groups. The post hoc result of the repetition measure indicated that the old‐age groups presented significantly higher values than the young group. Significant age effects were noted in total ICUs, ICU/s, MC scores, and ACs/min for macrostructural linguistics variables. Post‐hoc analyses of all four measures indicated that the young group performed significantly better than those peers in the old‐aged groups. The middle‐aged only significantly outperformed the old‐aged in total ICUs.

Education effects were obvious in most productivity measures (i.e., duration, total utterances, types, and tokens and tokens/min) and lexical diversity (i.e., VocD), in which the high‐education group had significantly higher values than the low‐education group. This phenomenon is also observed in producing information words (total ICUs) and MCs (MC scores).

#### 3.3.2 The Tortoise and Hare

Refer to Table 4. Significant age effects were found in the open‐to‐closed class ratio, total ICUs, ICUs/s, ICUs/utterance, MC scores, and ACs/min. Post‐hoc analyses revealed that the young group had significantly higher values than the old‐aged group. Regarding the use of open‐class words, the young group also showed significantly higher usage compared to the middle‐aged group.

**TABLE 4:** Means, standard deviations, and group comparisons^1^ of the narrative story – ‘The Tortoise and Hare’.

	18–39 years old group		40–59 years old group		> 60 years old group				
Variables	Low ed. (*N* = 24)	High ed. (*N* = 24)	Low ed. (*N* = 31)	High ed. (*N* = 17)	Low ed. (*N* = 24)	High ed. (*N* = 30)	Age main effect	Edu. main effect	Post‐hoc
Microstructural linguistics									
Duration (s)	54.75 (21.35)	65.83 (25.13)	73.90 (33.71)	77.47 (37.53)	56.42 (28.31)	82.90 (44.94)	3.594	2.114^*^	H > L^*^
Total utterance	21.38 (7.31)	30.38 (13.17)	32.64 (17.30)	33.61 (17.82)	23.67 (10.82)	34.91 (20.58)	3.598	2.228^*^	H > L^*^
MLU‐words	6.84 (0.99)	6.98 (1.00)	6.70 (1.19)	6.69 (0.91)	6.63 (1.17)	6.77 (0.98)	1.341	0.538	
Types	73.50 (19.50)	91.96 (25.64)	91.29 (30.80)	96.81 (32.62)	70.74 (24.21)	97.60 (36.68)	2.599	3.057^**^	H > L^**^
TTR	0.53 (0.07)	0.47 (0.07)	0.45 (0.07)	0.48 (0.07)	0.48 (0.07)	0.47 (0.08)	6.689	1.263	
Tokens	145.17 (50.88)	206.42 (83.31)	212.16 (96.81)	215.24 (96.91)	153.33 (75.24)	228.07 (118.52)	3.445	2.780^**^	H > L^**^
Tokens/min	161.69 (26.57)	187.43 (17.54)	173.23 (20.80)	172.25 (29.94)	164.81 (23.90)	169.37 (22.39)	3.282	2.035^*^	H > L^*^
Verbs/utterance	1.47 (0.31)	1.28 (0.19)	1.31 (0.37)	1.23 (0.31)	1.30 (0.31)	1.26 (0.23)	5.880	1.513	
Noun‐verb ratio	0.67 (0.19)	0.66 (0.16)	0.66 (0.18)	0.82 (0.18)	0.69 (0.18)	0.65 (0.17)	2.868	0.898	
O‐C ratio	1.37 (0.32)	1.01 (0.14)	0.94 (0.19)	1.17 (0.37)	1.02 (0.20)	1.02 (0.27)	11.927^**^	0.966	Y > M^**^; Y > O^**^
Retracing	0.88 (0.99)	0.54 (0.78)	0.61 (0.80)	1.24 (1.79)	1.08 (1.02)	0.83 (0.99)	1.857	0.481	
Repetitions	1.20 (1.80)	1.20 (1.35)	2.06 (1.39)	1.12 (1.17)	1.78 (2.02)	1.32 (1.44)	4.869	1.264	
VocD	49.07 (11.35)	48.90 (11.67)	48.12 (12.26)	53.16 (12.26)	43.21 (13.33)	50.50 (11.88)	1.010	1.585	
Macrostructural linguistics									
Total ICUs	20.54 (4.41)	22.46 (4.09)	21.19 (4.61)	23.53 (3.64)	17.21 (5.43)	20.10 (5.36)	11.775^**^	2.261^*^	Y > O^**^; M > O^**^; H > L^*^
ICUs/s	0.42 (0.16)	0.38 (0.14)	0.33 (0.12)	0.36 (0.14)	0.38 (0.13)	0.31 (0.15)	7.552^*^	0.905	Y > O^**^
ICUs/word	0.15 (0.05)	0.12 (0.05)	0.16 (0.05)	0.13 (0.05)	0.13 (0.05)	0.12 (0.05)	5.703	1.716	
ICUs/utterance	1.05 (0.34)	0.86 (0.35)	0.80 (0.36)	0.85 (0.35)	0.83 (0.32)	0.75 (0.35)	6.617^*^	1.146	Y > O^**^
MC scores	21.12 (4.04)	22.42 (2.93)	21.55 (4.07)	21.94 (3.70)	18.42 (5.63)	19.97 (4.13)	10.607^**^	0.671	Y > O^**^; M > O^**^
ACs/min	8.18 (3.37)	7.10 (2.83)	6.26 (2.35)	6.07 (2.39)	6.32 (2.94)	5.49 (2.99)	8.006^*^	1.718	Y > O^**^

*Note*: The values are listed in the order ‘mean (standard deviation)’.

Abbreviations: AC = accurate‐and‐complete main concept; ICU = information content unit; M = middle‐age; MC = main concept; MLU‐Words = mean length utterance (words); O = old‐age; O‐C ratio = open‐to‐closed class ratio; TTR = type‐token ratio, VocD = lexical diversity; Y = young‐age.

^1^ = non‐parametric tests (i.e., Kruskal–Wallis *H* test and Mann–Whitney *U* test) were performed because all the variables were non‐normally distributed.

* = *p* < 0.05,

** = *p* < 0.017.

Significant education effects were observed in duration, total utterances, types, tokens, tokens per minute, and total ICUs, with the high education group demonstrating significantly higher values than those in the low education group across all these measures.

### 3.4 Story Effect

Friedman test was performed to investigate whether the two stories are comparable to each other in each age and education subgroup, Kendall's *W* test using Chi‐square was used in calculating the effect size. Details of the result are listed in Table 5.

**TABLE 5:** Performance evaluation of stories by age and education subgroups.

	Age and education subgroups					
Variables	Y‐Low ed.	Y‐High ed	M‐Low ed.	M‐High ed.	O‐Low ed.	O‐ High ed.
Microstructural linguistics						
Duration (s)	2.130	0.429	4.800^*^; *W* = 0.123	2.225	0.182	5.828^*^; *W* = 0.161
Total Utterance	2.909	8.167^*^; *W* = 0.299	7.538^*^; *W* = 0.210	0.059	0.429	11.571^**^; *W* = 0.352
MLU‐Words	0.667	< 0.001	3.903^*^; *W* = 0.094	2.882	0.167	1.200
Types	3.522	4.167^*^; *W* = 0.132	2.613	0.250	0.167	1.690
TTR	8.167^*^; *W* = 0.299	2.667	9.323^*^; *W* = 0.268	7.118^*^; *W* = 0.360	0.043	10.800^**^; *W* = 0.327
Tokens	3.522	10.667^*^; *W* = 0.403	11.645^**^; *W* = 0.343	0.529	0.167	8.533^*^; *W* = 0.251
Tokens/min	2.667	8.167^*^; *W* = 0.299	7.258^*^; *W* = 0.202	1.471	0.667	0.533
Verbs/utterance	0.167	2.667	7.258^*^; *W* = 0.202	2.882	2.130	0.571
Noun‐verb ratio	0.167	0.167	0.290	7.118^*^; *W* = 0.360	0.167	< 0.001
O‐C ratio	1.500	2.667	0.032	0.529	4.167^*^; *W* = 0.133	0.133
Retracing	0.067	0.250	9.800^*^; *W* = 0.284	0.091	1.000	1.316
Repetitions	0.286	0.250	1.500	1.143	0.889	5.000^*^; *W* = 0.011
VocD	0.667	1.500	0.032	0.529	0.167	0.533
Macrostructural linguistics						
Total ICUs	18.182^**^; *W* = 0.716	19.174^**^; *W* = 0.757	22.533^**^; *W* = 0.694	17.000^**^; *W* = 0.941	2.667	19.200^**^; *W* = 0.606
ICUs/s	6.000^*^; *W* = 0.208	6.000^*^; *W* = 0.208	7.258^*^; *W* = 0.202	2.882	4.167^*^; *W* = 0.133	6.533^*^; *W* = 0.184
ICUs/word	0.667	2.667	7.258^*^; *W* = 0.202	1.471	4.167^*^; *W* = 0.133	3.333
ICUs/utterance	2.130	2.667	5.452^*^; *W* = 0.144	0.529	1.500	1.200
MC scores	19.174^**^; *W* = 0.757	23.000^**^; *W* = 0.917	20.161^**^; *W* = 0.618	17.000^**^; *W* = 0.941	6.545^*^; *W* = 0.231	19.200^**^; *W* = 0.607
ACs/min	8.167^*^; *W* = 0.299	6.000^*^; *W* = 0.208	5.452^*^; *W* = 0.144	2.882	3.522	5.828^*^; *W* = 0.161

*Note*: Kendall's *W* test was used to calculate the effect size; 0.1 indicates small effect; 0.3 indicates medium effect; and 0.5 indicates large effect.

Abbreviations: AC = accurate‐and‐complete main concept; ICU = information content unit; M = middle‐age; MC = main concept; MLU‐Words = mean length utterance (words); O = old‐aged group; O‐C ratio = open‐to‐closed class ratio; TTR = type‐token ratio; VocD = lexical diversity; Y = young‐aged group.

* = *p* < 0.05,

** = *p* < 0.001.

For those productivity measures, the two stories (i.e., The Boy Who Cried Wolf = S1; The Tortoise and Hare = S2) manifested differently in all subgroups. For example, the value of MLU‐word was found only significantly higher in the middle‐aged low‐education group in producing S1 than S2, with a small effect size (*χ* ^2^ = 3.903, *p* = 0.048, *W* = 0.094). Meanwhile, only the young‐aged, high‐education group demonstrated a significantly greater usage of types in S1 than S2 (*χ* ^2^ = 4.167, *p* = 0.041, *W* = 0.132). For the rest of the productivity measures that indicated significant differences, S1 showed a higher value than S2, yielding a small to medium effect size. Regarding syntactic complexity, S1 was found with higher verbs/utterance and open‐to‐closed class ratio than S2 in the middle‐aged low‐education group (*χ* ^2^ = 7.258, *p* = 0.007, *W* = 0.202) and the old‐aged low‐education group (*χ* ^2^ = 4.167, *p* = 0.041, *W* = 0.133), respectively, both indicating a small to medium effect size. However, S2 was observed with a higher noun‐verb ratio than S1 in the middle‐aged high‐education group with a medium effect size (*χ* ^2^ = 7.118, *p* = 0.008, *W* = 0.360). Retracing and repetition were found significantly higher in S1 than S2 in the middle‐aged low‐education group with a medium effect size (*χ* ^2^ = 9.800, *p* = 0.002, *W* = 0.284) and the old‐aged high‐education group with a small effect size (*χ* ^2^ = 5.000, *p* = 0.025, *W* = 0.011), respectively. Regarding lexical diversity, S1 showed significantly lower TTR than S2 in young‐aged low education (*χ* ^2^ = 8.167, *p* = 0.004, *W* = 0.299), middle‐aged low education (*χ* ^2^ = 9.323, *p* = 0.002, *W* = 0.268), middle‐aged high education (*χ* ^2^ = 7.118, *p* = 0.008, *W* = 0.360) and old‐aged high education groups (*χ* ^2^ = 10.800, *p* < 0.001, *W* = 0.327), all indicating a medium effect size.

The two stories were again not comparable to each other on most macrostructural linguistics variables. For example, S1 was observed with higher ICU than S2 in all subgroups with a large effect size except the old‐aged low‐education group. For ICUs/s, S1 indicated higher efficiency than S2 in all subgroups with small to medium effect size except the middle‐aged high‐education group. Meanwhile. S1 only indicated higher ICUs/utterance in the middle‐aged low‐education group (*χ* ^2^ = 5.452, *p* = 0.020, *W* = 0.144) than that of S2 with a small effect size. S1 demonstrated consistently higher total MC scores than S2 in all subgroups with medium to large effect sizes.

## 4 Discussion

This report on studying Cantonese narration stands out as one of the scarce reports that have successfully created an extensive norm for connected spoken language measurements. It accomplished this by analysing the spontaneous narrative discourse production of many native Cantonese‐speaking Chinese across different age groups and education levels. As mentioned in Kong et al. (2025), we emphasized ‘comprehensiveness’ and ‘representativeness’ in establishing normative references; this study extended the investigation to a genre of ‘narrative discourse’ that has not yet been explored. This normative reference would benefit clinical use and research studies on ageing and disordered spoken language, allowing the users to objectively evaluate a narrative spoken language sample by its age and education subgroup. The high inter‐rater reliability suggested consistency in scoring the spoken language samples, ensuring this normative reference's reliability.

The effect of healthy ageing was observed in both narrative stories in the present study but in a relatively inconsistent manner. By comparing the microstructural linguistics variables, some productivity and dysfluency measures were affected in one story but not another. It contradicted a stereotyped thought in Chinese that older people tend to tell a wavy, less fluent story than their younger counterparts, while younger people must have higher productivity and lexical diversity than older folks. Our findings further support the need for establishing normative references for discourse before estimating one's discourse ability. Furthermore, our results suggested some measures (i.e., open‐to‐closed class ratio, total ICUs, ICUs/s, MC scores, and ACs/mins) in storytelling appeared to be more sensitive to ageing.

Among all microstructural linguistics variables, only the open‐to‐closed class ratio was significantly influenced by age in both stories, in which the young‐aged group demonstrated higher usage of open‐class words than the middle‐ and old‐aged group. Open‐class words are often more semantically rich and diversified, which facilitates the construction of complex clauses and sentences, contributing to syntactic complexity. In contrast, excessive use of closed‐class words, often more abstract and general, can lead to a lack of specificity and detail in storytelling (Berman et al. 1994). Hence, the reducing of the open‐to‐closed ratio might be one of the key factors that older adults produced more ambiguous sentences (Sherratt and Bryan 2019), marking the spoken narratives with reduced complexity and coherence compared to younger adults (Babaei et al. 2019). It is noteworthy that the usage of open‐class words did not uniformly decline with increasing age (i.e., it was insignificant between middle‐aged and old‐aged groups in both stories), suggesting this ability may be preserved in participants aged between 40 and 71. This shared a similar pattern with the study conducted by Marini et al. (2025), who observed local and global coherence performance of narrative remained stable between the ages of 40 and 74.

In contrast, the age effect on macrostructural linguistics variables was more consistent. The total scores for ICUs, ICUs/s, MC scores, and ACs/min of both stories were reduced with ageing in a similar pattern. Our results indicated that informative unit production declines with increasing ages, in which the young age performed better than the middle‐aged, and the middle‐aged performed better than the old age group. It is also noted that younger people produced more thematic‐related concepts than their older counterparts, as reflected by the MC scores. The quantitative analysis of the macrostructural linguistics variables in this study agreed with Duong and Ska (2001) and Wright et al. (2014), who measured story propositions in story narratives and found age‐related declines in discourse MCs. ICUs/s and ACs/min reflected how efficiently a person conveyed an informative word and a concept. The old‐aged group was found with a reduced efficiency compared to the young‐aged group in both stories, suggesting the older adults tended to use more words or required more time to express the same amount of content as their young counterparts. This may also imply a change of cognitive abilities related to lexical concept extraction and selection or the use of compensatory strategies (e.g., sentence fillers) to gain more time to access relevant words and concepts (Schmitter‐Edgecombe et al. 2000).

Summarizing how macrostructural linguistics variables are affected by ageing, the results indicate a less organized, looser coherence and less efficient narrative ability in older age. Comparing across genres, age‐related decline in content‐based measures was also profound; for example, it was observed in descriptive discourse (Boucher et al. 2022) and sequential picture description (Kong et al. 2025). This phenomenon might further suggest the correlations of macrostructural linguistics measures with cognitive abilities, such as executive functions and metacognitive skills (Kavé and Goral 2017; Kintz et al. 2016).

Previous studies suggested lexical diversity as one of the microstructural linguistics variables more preserved in ageing (Fergadiotis et al. 2011; Kemper and Sumner 2001). This study performed VocD to analyse the lexical diversity of the narrative stories. The age effect was not observed in both stories across all age groups, suggesting no evidence of younger people being more privileged than older adults in lexical diversity. The result agreed with a similar study carried out by Fergadiotis et al. (2011), who also matched the participants in age and education level and found that age did not affect lexical diversity in story elicitation using a wordless picture book. Another possible reason for recording no apparent differences in lexical diversity between age groups might be the narrative task itself. Although the storybook was not provided during the formal recording in our study, probably the picture stimuli that we showed during the preparation stage sufficiently scaffolded the story structure and adequately promoted the vocabulary in younger adults, while the picture stimuli constrained the older people's productions, that is, from producing unrelated lexicons. Also, the selected narrative stories are well‐familiarized in the Chinese population, having been told in childhood. This might affect recalling similar story structure and vocabulary.

Another objective of the study is to examine the effect of education on storytelling. The education effect was observed in five productivity measures (i.e., duration, total utterances, types, tokens, and tokens per minute) and total ICUs of both stories. The result suggested that people in the higher education group tended to produce longer stories, using more uniform words and conveying more information than those in the lower education group. Our findings agreed with the research conducted by Cera and Silva (2020), who explored the impact of education levels on narrative storytelling skills and found higher levels of education were associated with greater information words. However, our findings only partially aligned with the study conducted by Le Dorze and BEDard (1998), in which the higher education group was observed with longer story duration, more open‐class words and types of words. In our study, education levels did not significantly influence the open‐closed word ratio. One possible explanation might be related to the difficulty level of the story context (i.e., unfamiliar story vs. fable story in this story). Further investigation is warranted to explore the context in which education effects can be manifested. Compared with the findings of Kong et al. (2025), the effects of education on storytelling were consistent with the other genres, in which higher education outperformed lower education in verbal productiveness and content informativeness.

‘The Boy Who Cried Wolf’ and ‘The Tortoise and Hare’ are both well‐familiarized fable stories in the local population. By tallying the number of microstructural linguistics measures affected by age, ‘The Boy Who Cried Wolf’ appeared to be more sensitive to ageing, while ‘The Tortoise and Hare’ remained relatively stable against ageing. For age effects on macrostructural linguistics measures, as well as the effect of education level, the two stories shared similar patterns. By comparing the performance of two stories by the same group of participants, our findings suggested that the two stories were not comparable to each other, as ‘The Boy Who Cried Wolf’ was observed with higher values in productivity, syntactic complexity, lexical diversity, dysfluency, informative words, and thematic concepts than ‘The Tortoise and Hare’. It was also noted that the performance of the two stories manifested differently across all subgroups. For example, the young‐aged and old‐aged low‐education groups performed relatively congruently across most microstructural linguistic parameters in both stories, while the middle‐aged low‐education and old‐aged high‐education groups indicated more variations. This further supports the need to address the interplay of age and education in discourse analysis even when the same type of story (i.e., a fable story in this study) is used for elicitation.

### 4.1 Clinical Implications

Discourse analysis provides many advantages in understanding one's communication abilities; it also allows comparisons over time (Kong 2022). This study expands the bank of normative data in Chinese aphasiology and provides normative references of narrative discourse that could be readily used in everyday clinical settings. Nevertheless, the data could be applied to the geriatric population, relating narrative skills to healthy ageing.

This story's normative data could be used to tap treatment effects. Treatments or activities for narrative story ability usually target relating events or experiences in temporal order; for example, the Novel Approach to Real‐life Communication: Narrative Intervention in Aphasia (Whitworth et al. 2015) and narrative training that addresses story grammar were usually implemented for mild types of aphasia, cognitive communication disorders, or social groups for the aged. Instead of using trained items for measurement, in which the treatment effect might be masked by the practice effect, the stories of this study could be used as ‘standardized,’ untrained tasks to objectively measure the treatment effect. This pre‐ and post‐test arrangement could reflect changes in one's performance comprehensively and objectively. Additionally, even if treatments are not directly targeted at discourse abilities (e.g., treatments targeted only on the word or sentence level), as story narrative could elicit more constrained content and provide common reference points for the speakers and listeners, this set of story norms could be used to detect if treatment effects have been generalized to higher level spoken language productions, as well as to predict one's communicating abilities in everyday life (Herbert et al. 2008). Notably, we suggested clinicians implement both stories or use the same story when conducting pre‐ and post‐tests, as the two stories are not comparable to each other in most micro‐ and macro‐structural linguistics measures. In addition, among all microstructural linguistic variables, our study showed verb/utterance, noun‐verb ratio and retracing behaved similarly regardless of age and education level in the two stories. Hence, keeping track of any decline of focus on actions and events or increase in going back for clarification or correction might be useful in monitoring discourse performance.

### 4.2 Limitations and Future Studies

The stories chosen in this study are fable stories, which might have fewer cognitive demands on subjects than using an unfamiliarized picture book for story elicitation. To a certain extent, the choice of fable stories limited the generalization of results to a broader sense of story narrative (i.e., telling an unfamiliar story). It is anticipated that some microstructural linguistics variables (e.g., lexical diversity measures and fluency measures), as well as the informative words and MC measures, might vary more in stories other than fable stories. Second, gender differences were not considered in our analysis. The effect of gender on spoken narrative is still debatable; for example, the study of Juncos Rebadan (1996) found a non‐significant gender effect and highlighted age and education as the major roles, while Davis et al. (2015) found women had higher accuracy of detail recall in social stories. Third, the presentation of stories to participants was not in random order, in which ‘The Boy Who Cried Wolf’ was presented first, followed by ‘The Tortoise and Hare,’ making us unable to exclude the possible effects induced by presentation order (e.g., participant engagement, priming effect for the subsequent story, etc.). Lastly, though the two stories were observed to be elicited differently by the same group of participants, the reasons contributing to the discrepancy have not yet been discovered. Additional analyses might investigate and compare the stories’ cognitive load, plot complexity, relatability to daily life or prior experiences and so forth, to find out the differences.

Future studies can extend the analysis to unfamiliarized story spoken discourse and consider gender as a covariance. New studies investigate how the newly developed normative data is being used in clinical settings, collect feedback, and evaluate to what extent the development of norms benefits clinical settings. This will grow the evidence for discourse studies and promote the development of discourse norm data in other populations (e.g., Mandarin‐speaking population).

## Conflicts of Interest

The authors declare no conflicts of interest.

## Supporting information




**Supporting Table 1**: Descriptive statistics of the narrative story ‐ “The Boy Who Cried Wolf”. **Supporting Table 2**: Descriptive statistics of the narrative story ‐ “The Tortoise and Hare”.

**Appendix 1a**: Checklist of “The Boy Who Cried Wolf” (Chinese version)
**Appendix 1b**: Checklist of “The Boy Who Cried Wolf” (English‐translated version)
**Appendix 2a**: Checklist of “The Tortoise and Hare” (Chinese version)
**Appendix 2b**: Checklist of “The Tortoise and Hare” (English‐translated version)
**Appendix 3**: Main concepts in Cantonese for narrative story ‐ The Boy Who Cried Wolf
**Appendix 4**: Main concepts in Cantonese for narrative story‐ The Tortoise and Hare

## Data Availability

The data that support the findings of this study are available from the corresponding author, AK, upon reasonable request.
