# Supplementary material for: Establishing Norms of Connected Speech Measures for Story‐Telling in Cantonese‐Speaking Adults
Source: Int J Lang Commun Disord. 2025 Sep 30;60(6):e70131. doi: 10.1111/1460-6984.70131 (PMC12483306; doi:10.1111/1460-6984.70131)
Supplement: Supplementary file 1 — Supporting Table 1: Descriptive statistics of the narrative story ‐ “The Boy Who Cried Wolf”. Supporting Table 2: Descriptive statistics of the narrative story ‐ “The Tortoise and Hare”. [file JLCD-60-0-s002.docx]

Supplementary Table 1.

*Descriptive statistics of the narrative story - “The Boy Cried Wolf”.*

| Variables | Mean(SD) | Median | Minimum | Maximum | Interquartile range | Skewness | Kurtosis | Normality^a^ |
| --- | --- | --- | --- | --- | --- | --- | --- | --- |
| Microstructural linguistics | | | | | | | | |
| Duration (sec) | 76.71 (41.57) | 65.00 | 31.00 | 196.00 | 45.00 | 1.35 | 1.29 | <.001 |
| Total Utterance | 34.79 (21.11) | 28.50 | 12.00 | 99.00 | 20.00 | 1.58 | 2.06 | <.001 |
| MLU-Words | 6.81 (1.03) | 6.59 | 5.33 | 9.12 | 1.31 | 0.62 | -0.32 | .003 |
| Types | 93.59 (33.51) | 86.50 | 48.00 | 186.00 | 42.00 | 1.08 | 0.96 | <.001 |
| TTR | 0.44 (0.09) | 0.44 | 0.30 | 0.61 | 0.13 | 0.23 | -0.75 | .200 |
| Tokens | 232.67 (136.25) | 202.50 | 37.00 | 748.00 | 135.00 | 1.72 | 3.38 | <.001 |
| Tokens/min | 183.16 (31.42) | 184.70 | 124.18 | 245.45 | 47.25 | -0.02 | -0.69 | .200 |
| Verbs/utterance | 1.41 (0.31) | 1.37 | 1.00 | 2.27 | 0.38 | 0.93 | 0.46 | .005 |
| Noun-verb ratio | 0.66 (0.12) | 0.67 | 0.45 | 0.89 | 1.56 | 0.03 | -0.41 | .200 |
| O-C ratio | 1.07 (0.21) | 1.05 | 0.76 | 1.56 | 0.33 | 0.56 | -0.52 | .015 |
| Retracing | 1.23 (1.46) | 1.00 | 0.00 | 5.00 | 2.00 | 1.12 | 0.31 | <.001 |
| Repetitions | 2.02 (2.25) | 1.00 | 0.00 | 8.00 | 3.00 | 1.23 | 0.71 | <.001 |
| VocD | 49.27 (12.52) | 47.80 | 29.78 | 76.51 | 18.76 | 0.42 | -0.53 | .024 |
| Macrostructural linguistics | | | | | | | | |
| Total ICUs | 27.79 (7.02) | 29.00 | 5.00 | 40.00 | 9.00 | -0.85 | 0.77 | .017 |
| ICUs/sec | 0.43 (0.18) | 0.42 | 0.08 | 0.94 | 0.22 | 0.44 | 0.93 | .086 |
| ICUs/word | 0.14 (0.05) | 0.14 | 0.03 | 0.30 | 0.63 | 0.46 | 0.19 | .037 |
| ICUs/utterance | 0.98 (0.42) | 0.92 | 0.13 | 2.42 | 0.53 | 0.48 | 0.23 | .078 |
| MC scores | 27.55 (5.70) | 29.00 | 6.00 | 33.00 | 7.00 | -1.06 | 2.64 | <.001 |
| ACs/min | 8.53 (2.24) | 9.00 | 0.00 | 11.00 | 2.25 | -1.32 | 1.74 | .082 |

*Note1.* MLU-Words = mean length utterance (words), TTR = type-token ratio, O-C ratio = open-to-closed class ratio, VocD=lexical diversity, ICU = information content unit,

MC = main concept, AC = accurate-and-complete main concept

*Note2.* ^a^Normality verified by Kolmogorov-Smirov test, *p* < .05 infers non-normally distributed data.

Supplementary Table 2.

*Descriptive statistics of the narrative story - “Tortoise and Hare”.*

| Variables | Mean(SD) | Median | Minimum | Maximum | Interquartile range | Skewness | Kurtosis | Normality^a^ |
| --- | --- | --- | --- | --- | --- | --- | --- | --- |
| Microstructural linguistics | | | | | | | | |
| Duration (sec) | 68.95 (34.30) | 58.00 | 28.00 | 152.00 | 44.00 | 1.06 | 0.19 | <.001 |
| Total Utterance | 29.68 (6.20) | 26.00 | 11.00 | 72.00 | 18.00 | 1.91 | 0.70 | <.001 |
| MLU-Words | 6.77 (6.60) | 6.59 | 5.06 | 8.92 | 1.46 | 0.43 | -0.63 | .011 |
| Types | 87.41 (31.04) | 82.00 | 43.00 | 162.00 | 40.00 | 0.76 | -0.00 | .001 |
| TTR | 0.48 (0.08) | 0.47 | 0.31 | 0.70 | 0.12 | 0.18 | -0.00 | .040 |
| Tokens | 194.64 (94.88) | 176.00 | 79.00 | 409.00 | 120.00 | 0.91 | -0.030 | <.001 |
| Tokens/min | 171.22 (24.32) | 170.84 | 126.09 | 216.39 | 36.34 | 0.01 | -0.79 | .014 |
| Verbs/utterance | 1.31 (0.30) | 1.26 | 0.87 | 2.04 | 0.38 | 0.73 | 0.09 | .010 |
| Noun-verb ratio | 0.68 (0.18) | 0.67 | 0.40 | 1.04 | 0.28 | 0.27 | -0.88 | .047 |
| O-C ratio | 1.08 (0.29) | 1.02 | 0.69 | 1.82 | 0.32 | 1.13 | 0.92 | <.001 |
| Retracing | 0.85 (1.05) | 0.50 | 0.00 | 4.00 | 1.00 | 1.25 | 1.16 | <.001 |
| Repetitions | 1.55 (1.67) | 1.00 | 0.00 | 6.00 | 2.00 | 1.07 | 0.46 | <.001 |
| VocD | 48.68 (12.25) | 46.80 | 27.11 | 73.10 | 16.07 | 0.27 | -0.59 | .027 |
| Macrostructural linguistics | | | | | | | | |
| Total ICUs | 20.69 (5.00) | 21.00 | 3.00 | 31.00 | 7.00 | -0.52 | 0.20 | .002 |
| ICUs/sec | 0.35 (0.15) | 0.34 | 0.07 | 0.79 | 0.20 | 0.62 | -0.17 | .007 |
| ICUs/word | 0.12 (0.05) | 0.12 | 0.03 | 0.24 | 0.07 | 0.54 | -0.48 | .004 |
| ICUs/utterance | 0.85 (0.36) | 0.76 | 0.19 | 1.82 | 0.56 | 0.47 | -0.60 | <.001 |
| MC scores | 20.85 (4.31) | 21.00 | 2.00 | 27.00 | 6.00 | -0.98 | 1.67 | <.001 |
| ACs/min | 6.54 (2.93) | 6.00 | 0.00 | 15.00 | 3.33 | 0.73 | 0.39 | .005 |

*Note1.* MLU-Words = mean length utterance (words), TTR = type-token ratio, O-C ratio = open-to-closed class ratio, VocD=lexical diversity, ICU = information content unit,

MC = main concept, AC = accurate-and-complete main concept

*Note2.* ^a^Normality verified by Kolmogorov-Smirov test, *p* < .05 infers non-normally distributed data
