# Supplementary material for: Establishing Norms of Connected Speech Measures for Story‐Telling in Cantonese‐Speaking Adults
Source: Int J Lang Commun Disord. 2025 Sep 30;60(6):e70131. doi: 10.1111/1460-6984.70131 (PMC12483306; doi:10.1111/1460-6984.70131)
Supplement: Supplementary file 2 — Appendix 1a: Checklist of “The Boy Who Cried Wolf” (Chinese version) Appendix 1b: Checklist of “The Boy Who Cried Wolf” (English‐translated version) Appendix 2a: Checklist of “The Tortoise and Hare” (Chinese version) Appendix 2b: Checklist of “The Tortoise and Hare” (English‐translated version) Appendix 3: Main concepts in Cantonese for narrative story ‐ The Boy Who Cried Wolf Appendix 4: Main concepts in Cantonese for narrative story‐ The Tortoise and Hare [file JLCD-60-0-s001.docx]

Appendix 1a.

Checklist of “The Boy Who Cried Wolf” (Chinese version)

| Scenario | ICUs (frequency) | | | | | MCs (frequency) |
| --- | --- | --- | --- | --- | --- | --- |
|  | Subjects | Places | Objects | Actions | Others |  |
| 1. Setting | 1. 牧羊人/牧童/小朋友/細路仔/男仔/看羊/放/牧羊嘅小朋友/細路仔/[-牧羊] (90%) | 1.山 (46.67%) | 1. [牧-] 羊 (92.67%) 2. [食-] 草 (25.33%) | 1. 放/牧/牧養/睇/看/帶/趕 [-羊] (92.67%) 2. 上 [-山] (24.67%) 3. 食 [-草] (23.33%) | 1. 成日/日日/每一日 [-帶] (25.33%) 2. 有一日/從前/好耐以前 (27.33%) | MC1. 小朋友/牧童/小牧羊人/牧羊嘅小朋友 **牧/看/睇/放/趕** 羊 OR 小朋友/牧童/小牧羊人/牧羊嘅小朋友 **帶** 羊上山  (86.00%) |
| 2. Inferring why the boy cried wolf | 1.佢/小朋友 [-覺得] (76.67%) |  |  | 1. 覺得 [-好悶] (49.33%) | 1. 非常/好悶/悶過頭/苦悶/無聊得滯/好無聊/百無聊賴(71.33%) | MC2.佢/個小朋友 (**覺得**) 好悶/好無聊  (70.67%) |
| 3. The boy lied | 1. 佢 [-嗌] (68.00%) |  |  | 1. 叫/嗌/話/講[-狼來了] (94.67%) | 1. [叫/嗌/話/講-]「狼來了」/「有狼嚟啦」(92.67%) 2. 大/大聲/猛/博命 [-叫/嗌] (30.67%) | MC3. (佢) **嗌/叫/話/講** 「狼來了」/「有狼嚟啦」/山上有狼  (92.67%) |
| 4. The reaction of the villagers | 1. 村民/大人/山下面啲人/農夫[-上] (94%)  2. [幫/救-] 小朋友/牧童/佢 (28.00%) | 1.山 (26.00%) | 1. [攞-] 武器/工具/斧頭/棍/鋤頭/耕具/剷/木 (36.67%) 2. [趕/打/捉/殺-] 狼 (36.00%) | 1. 跑上/走上/衝上/上去/上來 (88.67%) 2. 攞/帶/拎/揸 [-武器] (34.00%)  3. 幫/救 [-牧童] (34.00%)  4. 趕/捉/打/殺 [-狼] (39.33%) |  | MC4. 村民/農夫/啲人/大人就 **上嚟**/**上去**  OR  村民/農夫/啲人/大人就 **跑**上 山  (87.33%)  **AND**  MC5. (村民/農夫/啲人/大人) **趕/打/捉**/**殺** 狼  OR  (村民/農夫/啲人/大人) **幫/救** 牧童/佢  (71.33%) |
| 5. The truth | 1. 佢/小朋友/細路仔/牧童/男仔 [-講大話] (40.67%) |  | 1. [無-] 狼 (27.33%) | 1. [牧童-] 講大話/呃/欺騙/整蠱(39.33%) 2. 發現/發覺 (28.00%) | 1. 點知 (42.67%) 2. 原來/根本 (38.67%) 3. 無 (33.33%) | MC6. 點知/原來 牧童/佢/ 個小朋友 **講大話**  OR  點知/原來牧童/佢/個小朋友 **呃/欺騙/整蠱** 人 OR  點知/原來 ﻿(村民) **發現** 無 狼/﻿乜都冇  (71.33%) |
| 6. Real wolf came | 1. 狼 [-嚟/出現] (90.00%) |  | 1. [食-] 羊 (22.67%) | 1. [狼-] 嚟/出現 (89.33%) 2. 食/咬/攻擊 [-羊] (22.00%) | 1. 點知/但是 (26.00%) 2. 有一次/後來/之後/跟住/下次/結果/終於/卒之(71.33%) 3. 真係 (88.67%) | MC7. 有一日 狼 真係 **嚟/來/出現**  (89.33%) |
| 7. Reaction of the boy on wolf coming | 1. 佢/小朋友/細路仔/牧童/牧羊人/個男仔 [-嗌] (79.33%) |  |  | 1. [牧童-] 嗌/叫/話 (81.33%) | 1.「狼來了」/「有狼呀」(56.67%) 2. 大/大聲/博命/猛 [-嗌/叫] (26.67%) | MC8. 牧羊人/佢/小朋友/牧童 **嗌** /**大叫﻿** 「狼來了！」/「救命呀！」  (65.33%) |
| 8. Reaction of the villagers on wolf coming | 1. 村民/大人/農夫/啲人 [-救/幫] (66.00%) 2. 冇人 [-救/幫] (28.00%) 3. [信/救/理-] 佢/小朋友 (69.33%) |  |  | 1. [冇人-] 救/幫/理/信 (27.33%) 2. [村民-] 唔救/幫/理/信 (60.67%) | 1. 點知/但係/不過 (38.00%) | MC9. 大人/村民/山下面嘅人 **唔信**/**理**/**幫**/**救** 佢  OR  冇人 **信/理/幫/救** 佢  (81.33%) |
| 9. Consequences of the event | 1. 啲羊 (49.33%) 2. 狼 [-食咬/捉/殺] (37.33%) |  | 1. [食晒/咬死-] 啲羊 (37.33%) | 1. [羊-] 被狼食晒 (47.33%) 2. [狼-] 食/咬/捉/殺晒 (39.33%) | 1. 所以/於是/卒之/最後/結果 (42.67%) | MC10. 啲羊 被狼**食**晒  OR  狼 **食**/**捉**/**咬**/**殺**晒 啲羊  (84.67%) |
| 10. Reaction of the boy on the consequence | 1. 佢/小朋友/細路仔/牧童/牧羊人/個男仔[-返/傷心] (78.66%) | 1. [返-] 屋企 (30.33%) 2. [返-] 村 (25.33%) |  | 1. [牧童-] 返 (30%) | 1. [牧童-] 傷心/難過/唔開心/失望/無奈/後悔/內疚(53.33%) | MC11. 個 牧童 **返** 屋企  OR  個 牧童 好 **傷心**  (71.33%) |

Appendix 1b.

Checklist of “The Boy Who Cried Wolf” (English-translated version)

| Scenario | ICUs (frequency) | | | | | MCs (frequency) |
| --- | --- | --- | --- | --- | --- | --- |
|  | Subjects | Places | Objects | Actions | Others |  |
| 1. Setting | 1. Shepherd/ the shepherd boy/child/ The child who herded sheep [-herded] (90.00%) | 1.hill (46.67%) | 1. [herd-] sheep (92.67%) 2. [eat-] grass (25.33%) | 1. leading/ keeping/ watching [-sheep] (92.67%) 2. going up [-hill] (24.67%) 3. to eat [-grass] (23.33%) | 1. Everyday [-lead] (25.33%) 2. One day/ Once upon a time/ Long ago (27.33%) | MC1.  A shepherd boy/The child who herded sheep/The child/The boy was **herding/ leading/ keeping/ watching** his sheep OR A shepherd boy/The child who herded sheep/The child was **leading** his sheep to the hill  (86.00%) |
| 2. Inferring why the boy cried wolf | 1.He/ The child [-felt] (76.67%) |  |  | 1. felt [-bored] (49.33%) | 1. bored/ lame (71.33%) | MC2.  The child/He (felt/was) **bored/ lame**.  (70.67%) |
| 3. The boy lied | 1. He [-shouted] (68.00%) |  |  | 1. cried/ shouted/ said [-“Wolf!”] (94.67%) | 1. [cried/ shouted/ said] “Wolf has come!” “There’s wolf!” (92.67%) 2. loudly/ desperately [-cried/ shouted] (30.67%) | MC3.  (He) **cried/ shouted** “Wolf!”/ “Wolf has come!”/ “There’s wolf!”  (92.67%) |
| 4. The reaction of the villagers | 1. Villagers/ adults/ those people who lived on hillside/ farmers [-ran up] (94.00%)  2. [helped/saved-] the child/ the shepherd boy/ him (28.00%) | 1.hill (26.00%) | 1. [took-] weapons/ tools/ axe/ stick /hoe / shovel/ wood (36.67%) 2. [drove away/ hit-] the wolf (36.00%) | 1. came up/ran up (88.67%) 2. took/ brought/ carried [-weapon] (34.00%)  3. helped/ saved [-the child] (34.00%)  4. drove away/ caught/ hit [-wolf] (39.33%) |  | MC4.  Villagers/Farmers/  Those people/Adults **came**.  OR  Villagers/Farmers/  Those people/Adults **ran up** the hill.  (87.33%)  **AND**  MC5.  (Villagers/Farmers/  Those people/Adults) **drove away/caught/**  **hit** the wolf.  OR  (Villagers/Farmers/  Those people/Adults) **saved/helped** the shepherd boy/him.  (71.33%) |
| 5. The truth | 1. He/ the child/ the shepherd boy/ the boy [-told lie/ tricked/ lied] (40.67%) |  | 1. [no-] wolf (27.33%) | 1. [the shepherd boy-] told lie/ tricked/ lied. (39.33%) 2. found [-no wolf] (28.00%) | 1. but (42.67%) 2. In fact (38.67%) 3. no/nothing (35.82%) | MC6.  But, the shepherd boy **lied**. OR  But, the shepherd boy **tricked** the people.  OR But (the villagers ) **found/** nothing**/**no wolf.  (71.33%) |
| 6. Real wolf came | 1. The wolf [-came/ appeared] (90.00%) |  | 1. [ate-] the sheep (22.67%) | 1. [wolf-] came/ appeared (89.33%) 2. ate/ bit/attacked [-sheep] (22.00%) | 1. but (26.00%) 2. one day/ then/ after that/ finally (71.33%) 4. really (88.67%) | MC7.  One day, the wolf really **came/ appeared**.  (89.33%) |
| 7. Reaction of the boy on wolf coming | 1. He/ the child/ the shepherd boy/ the boy [-cried/shouted] (79.33%) |  |  | 1. [the shepherd boy-] cried/ shouted/ said /asked (81.33%) | 1. [cried/ shouted]-“Wolf!”/ “Wolf has come!” (56.67%) 2. loudly/ desperately [-cried/ shouted] (26.67%) | MC8.  He/The child/ The shepherd boy **cried/ shouted** “Wolf!”/ “Help!”  (65.33%) |
| 8. Reaction of the villagers on wolf coming | 1. Villagers/ farmers/ adults/ those people [-saved/ helped] (66.00%) 2. Nobody [-believed/saved/helped/ cared about] (28.00%) 3. [believed/ saved/ helped/cared about] him/ the child (69.33%) |  |  | 1. [Nobody-] saved/ helped / cared about/ believed (27.33%) 2. [Villagers-] did not help/believe/ save/ care about (60.67%) 3. [Villagers-] thought (29.33%) | 1. but (38.00%) | MC9.  The villagers/adults/ Those people **ignored** the shepherd boy. OR  Nobody **believed/ helped/saved/cared about** him.  (81.33%) |
| 9. Consequences of the event | 1. The sheep [-were eaten] (49.33%) 2. Wolf [-ate] (37.33%) |  | 1. [ate/ bit/ attacked-] the sheep (37.33%) | 1. [sheep-] were eaten by the wolf (47.33%) 2. [wolf-] ate/ bit/ caught (39.33%) | 1. therefore/ at last/ finally/ in the end (42.67%) | MC10. The sheep were **eaten** by the wolf.  OR  The wolf **ate/bit/ caught/killed** all the sheep.  (84.67%) |
| 10. Reaction of the boy on the consequence | 1. He/ the child/ the shepherd boy/ the boy [-went/sad] (78.66%) | 1. [went-] home (30.33%)  2. [went-] village (25.33%) |  | 1. [shepherd boy-] went (30%) | 1. [shepherd boy-] disappointed/ regret/ resigned/ guilt (53.33%) | MC11. The shepherd boy **went** home.  OR  The shepherd boy (felt/was) **sad**.  (71.33%) |

Appendix 2a.

Checklist of “The Tortoise and Hare” (Chinese version)

| Scenario | ICUs (frequency) | | | | | MCs (frequency) |
| --- | --- | --- | --- | --- | --- | --- |
|  | Subjects | Places | Objects | Actions | Others |  |
| 1. Setting | 1. 免仔 [-賽] (96.00%) 2. 烏龜 [-賽] (96.00%) | 1. 森林 (58.67%) | 1. [舉辦/參加/鬥-] 運動會/比賽/賽跑(30.67%) | 1. [龜兔-]賽跑 (39.33%)  2. 比賽/鬥/參加/進行/相約 [-賽跑] (30.00%) 3. 舉行/舉辦/進行 [-運動會] (28.00%) | 1. 同/同埋 (68.00%) 2. 從前/有一日 (45.33%) | MC1. 兔仔 同埋 烏龜 **賽跑**  OR  兔仔 同埋 烏龜 **參加/進行/鬥/** 比賽/跑步/賽跑  (67.33%) |
| 2: The hare took the lead at the beginning | 1. 兔仔 [-領先/跑] (69.33%) |  | 1. [拋離/超越-]烏龜(20.67%) | 1. [兔仔-] 領先/跑/跑出/拋離/超越 [-烏龜](66.67%) | 1. [跑/兔仔-]快過/快啲/快好多/第一 (53.33%) 2. 一開始/首先/開始時/起初 (57.33%) | MC2. 兔仔 (**跑**得) 快好多/快過烏龜  OR 兔仔 **領先/拋離** 烏龜  (69.33%) |
| 3:Inferring why the hare took rest | 1.兔仔 [-見/諗/覺得/發現/發覺] (92.00%) 2. 烏龜 [-追唔上/未到/慢] (64.67%) |  |  | 1. 見/見唔到/發現/發覺/諗/覺得 [-烏龜] (78.00%) 2. [烏龜-] 追唔上/跟唔上/未到 (50.67%) | 1. [行/走/跑/爬/烏龜-] 咁慢 (26.67%) | MC3. 兔仔 諗住/覺得 **蠃**梗  OR  兔仔 好驕傲  OR  兔仔 諗住/覺得 烏龜 有排**未到** OR 兔仔 諗住/覺得 烏龜 (跑得)咁慢  (76.67%) |
| 4. The hare took rest | 1. 兔仔 [瞓-] (64.67%) | 1. [兔仔] 樹下/樹度/樹上/樹林/路上/途中/終點前 (35.33%) |  | 1. [兔仔-]瞓覺/瞓一陣/休息一陣 (96.67%) | 1. 跟住/於是/所以 (30.00%) | MC4. (兔仔) **瞓**咗**覺**/**瞓**一陣/**休息**一陣  (96.67%) |
| 5. The tortoise passed the hare | 1. 烏龜 [-超越] (62.67%) |  | 1. [追上/超越-]兔仔/佢 (44.00%) | 1. 追上/趕過/超越/領先/爬到/行過 [-兔仔] (61.33%) |  | MC5. 烏龜 **追上/領先**  OR 烏龜 **趕過/超越/爬過/行過** 兔仔  (60.67%) |
| 6. The tortoise kept going | 1. 烏龜[-行/爬/跑] (64.00%) | 1. [爬/行-] 終點 (24.00%) |  | 1. [烏龜-] 行/爬/跑 (67.33%) | 1. 繼續/一直/不斷/喺噉/一步一步 (48.67%) | MC6. (烏龜) 不斷**行/爬/跑**  OR  (烏龜) **跑**向 終點  (67.33%) |
| 7. The hare woke up | 1. 兔仔 [-醒/起身] (62.00%) |  |  | 1. [兔仔-]瞓醒/ 醒/起身 (62.00%) 2. 跑/追/行/走去 (36.00%) |  | MC7. 兔仔 **起身/瞓醒**    (61.33%) |
| 8. The result of the race | 1. 烏龜 [-到達/蠃] (88.00%) | 1. [到達-] 終點 (36.67%) | 1. [得到/攞-] 冠軍/第一名/金牌 (27.33%) | 1. 先到/到達/衝過/跑到 [-終點] (37.33%) 2. [烏龜-] 蠃/勝出 (28.00%) 3. 攞/拎/得到/成為 [-冠軍/第一名] (24.67%] | 1. 結果/最終/最後/原來/但是 (40.00%) 2. 已經 (35.33%) | MC8. 烏龜 **先到/到達/衝過/跑到** 終點  OR 烏龜 **攞到/拎到/得到/成為** 冠軍/第一名/金牌  OR 烏龜 **蠃**了/**勝出**  (88.00%) |

Appendix 2b.

Checklist of “The Tortoise and Hare” (English-translated version)

| Scenario | ICUs (frequency) | | | | | MCs (frequency) |
| --- | --- | --- | --- | --- | --- | --- |
|  | Subjects | Places | Objects | Actions | Others |  |
| 1. Setting | 1. Hare [-race] (96.00%) 2. Tortoise [-race] (96.00%) | 1. forest (58.67%) | 1. [join/compete/  hold-] competition/ running/athletic meet (30.67%) | 1. [Tortoise & Hare-] race (39.00%)  2. join/ compete [-competition/ running] (30.00%) 3. hole [-athletic meet] (28.00%) | 1. and (68.00%) 2. Once upon a time/ One day/ Once (45.33%) | MC1.  Tortoise and hare **raced**  OR  Tortoise and hare **joined/ competed** a competition/ running match  (67.33%) |
| 2: The hare took the lead at the beginning | 1. Hare [-leading/ running] (69.33%) |  | 1. [surpassed/ overtook-] tortoise (20.67%) | 1. [hare-] led/ surpassed/ran/ overtook [-tortoise](66.67%) | 1. [ran/hare-] faster/ faster than/ the first (53.33%) 2. At the beginning/ At first (57.33%) | MC2. The hare (**ran)** faster than the tortoise /the first/so fast  OR  The hare **surpassed/ overtook** the tortoise  (69.33%) |
| 3:Inferring why the hare took rest | 1.Hare [thought/ found] (92.00%) 2. Tortoise [-not arrived/ slow] (64.67%) |  |  | 1. thought/found/ did not see [-tortoise] (78.00%) 2. [tortoise-] not arrived/ could not keep up (50.67%) | 1. [crawled/ran/  walked/tortoise-] slow (26.67%) | MC3.  ﻿The hare thought (he) would **win** / The hare was **arrogant**  OR  ﻿The hare thought the tortoise would **not arrive** shortly  OR  ﻿The hare thought the tortoise (ran) too **slow**  (76.67%) |
| 4. The hare took rest | 1. Hare [slept-] (64.67%) | 1. [Hare] tree/  runway/roadside (35.33%) |  | 1. [Hare-] slept/ rested(96.67%) | 1. So/Then/  Therefore (30.00%) | MC4.  (The hare) **slept/ rested**  (96.67%) |
| 5. The tortoise passed the hare | 1. Tortoise [-surpassed] (62.67%) |  | 1. [surpassed/ overtook-] Hare (44.00%) | 1. surpassed/ overtook/passed/  led [-hare] (61.33%) |  | MC5.  The tortoise **caught up**  OR  The tortoise **passed/ overtook** the hare  (60.67%) |
| 6. The tortoise kept going | 1. Tortoise[-crawled/ ran/ walked] (64.00%) | 1. [crawled/ran/  walked-] destination (24.00%) |  | 1. [Tortoise-] ran/ walked/ crawled (67.33%) | 1. continually (48.67%) | MC6.  (The tortoise) **ran/ walked/ crawled** continually  OR  (The tortoise) ﻿ran towards the  destination  (67.33%) |
| 7. The hare woke up | 1. Hare [-woke] (62.00%) |  |  | 1. [Hare-] woke up (62.00%) 2. [Hare-] ran/ walked/ went (36.00%) |  | MC7.  The hare **woke** up  (61.33%) |
| 8. The result of the race | 1. Tortoise [-won/ arrived] (88.00%) | 1. [arrived/ran to-] destination (36.67%) | 1. [got/received/ became-] champion/ gold medal/ the first (27.33%) | 1. arrived/ran to [-destination] (37.33%) 2. [tortoise-] won (28.00%) 3. got/received/ became [-champion/ the first] (24.67%) | 1. Finally/ At last/ But (40.00%) 2. already (35.33%) | MC8.  The tortoise **arrived/ ran** to the destination  OR  The tortoise **got/received/ became** the champion/the first/gold medal  OR  The tortoise **won**  (88.00%) |

Appendix 3

Main concepts in Cantonese for narrative story- The Boy Who Cried Wolf

The main verb for each main concept is **bolded**. All the essential information within a main concept is underlined.

| **Narrative Story (The Boy Who Cried Wolf)** | | |
| --- | --- | --- |
| MC | Version |  |
| 1 | Version 1 | A shepherd boy was **herding** his sheep  牧童 **看** 羊  *muk6 tung4*  ***hon1***  *joeng4* |
|  | Version 2 | A shepherd boy was **leading** his sheep to the hill  牧童 **帶** 羊 上 山  *muk6 tung4****daai3****joeng4 soeng5 saan1* |
| 2 | - | The shepherd boy was **bored**/**lame**  個 牧童 好 **悶**/**無聊**  *go3 muk6 tung4 hou2* ***mun6****/****mou4 liu4*** |
| 3 | - | (He) **cried** “Wolf!”  (佢) 就 **嗌** 「狼來了!」  *(keoi5) zau6* ***aai3****「 long4 loi4 liu5!*」 |
| 4 | Version 1 | Villagers **came**  村民 就 **上嚟**/**上去**  *cyun1 man4 zau6* ***soeng5 lai4/soeng5 heoi3*** |
|  | Version 2 | Villagers **ran** up the hill  村民 就 **跑** 上 山  *cyun1 man4 zau6* ***paau2*** s*oeng5 saan1* |
| 5 | Version 1 | (Villagers) **drove away** the wolf  (村民) **趕** 狼  *(cyun1 man4)****gon2*** *long4* |
|  | Version 2 | (Villagers) **saved/helped** the shepherd boy  (村民) **幫/救** 個 牧童  *(cyun1 man4)****bong1/gau3*** *go3 muk6 tung4* |
| 6 | Version 1 | But the shepherd boy **lied**  點知/原來 牧童 **講大話**  *dim2 zi1/jyun4 loi4 muk6 tung4* ***gong2 daai6 waa6*** |
|  | Version 2 | But the shepherd boy **tricked** the people  點知/原來 牧童 **呃** 人  *dim2 zi1/jyun4 loi4 muk6 tung4* ***aak1*** *jan4* |
|  | Version 3 | But (the villagers) **found** nothing**/** no wolf  點知/原來 (村民) **發現** 乜都冇/ 無 狼  *dim2 zi1/jyun4 loi4 (cyun1 man4)* ***faat3 jin6*** *mat1 dou1 mou5/ mou4 long4* |
| 7 | - | One day, the wolf really **appeared**  有一日， 狼 真係 **出現**  *jau5 jat1 jat6, long4 zan1 hai6* ***ceot1 jin6*** |
| 8 | - | The shepherd boy **shouted/cried** “Wolf!”/ “Help!”  牧童 **嗌**/大**叫**「狼來了！」/「救命呀！」  *muk6 tung4* ***aai3****/daai6* ***giu3*** 「*long4 loi4 liu!」/「gau3 ming6 aa1!」* |
| 9 | Version 1 | The villagers **ignored** the shepherd boy  村民 **唔理** 個 牧童  *cyun1 man4* ***m4 lei5*** *go3 muk6 tung4* |
|  | Version 2 | Nobody **cared about** the shepherd boy  冇人 **理** 個 牧童  *mou5 jan4* ***lei5****go3 muk6 tung4* |
| 10 | Version 1 | The sheep were **eaten** by the wolf  啲 羊 被 狼 **食**晒  *di1 joeng4 bei6 long4* ***sik6*** *saai3* |
|  | Version 2 | The wolf **ate** all the sheep  狼 **食**晒 啲 羊  *long4* ***sik6*** *saai3 di1 joeng4* |
| 11 | Version 1 | The shepherd boy **went** home  個 牧童 **返** 屋企  *go3 muk6 tung4* ***faan2*** *nguk1 kei5* |
|  | Version 2 | The shepherd boy was **sad**  個 牧童 好 **傷心**  *go3 muk6 tung4 hou2* ***soeng1 sam1*** |

Lexical items that are commonly accepted as alternatives in “The Boy Who Cried Wolf”

| MC | Essential | Alternatives |
| --- | --- | --- |
| 1，6，8 | 牧童 | 小朋友，牧羊人，牧羊嘅小朋友，細路仔/細路 |
| 1 | 看 [-羊] | 牧，睇，放，趕 |
| 2，5，9 | 牧童 | 小朋友，小牧羊人，牧羊嘅小朋友，細路仔/細路，佢（with proper referent） |
| 3 | [牧童-] 嗌 | 叫，話，講 |
| 3，8 | [嗌-] 「狼來了」 | 「有狼嚟啦」，「山/山上/山度有狼」，「有狼呀」 |
| 4，5，9 | 村民 | 農夫，大人，啲人，山下面嘅人 |
| 4 | 跑上 [-山] | 衝上，趕上，上 |
| 5 | [村民-] 趕 | 打，捉，殺 |
| 6 | [牧童-] 呃 | 欺騙，整蠱 |
| 7 | [狼-] 出現 | 嚟，嚟咗 |
| 7 | [有一次] | 有一日，後來，之後，跟住，隔咗一陣/排，終於/卒之 |
| 8 | [嗌-]「救命呀」 | 「救我呀」 |
| 9 | [村民/冇人-] 理 | 信，幫，救 |
| 10 | [狼-] 食 | 捉，咬，殺 |
| 11 | [返-] 屋企 | 村 |
| 11 | [牧童-] 傷心 | 難過，唔開心，失望，慘，後悔，內疚，無奈，黯然 |

Appendix 4

Main concepts in Cantonese for narrative story- The Tortoise and Hare

The main verb for each main concept is **bolded**. All the essential information within a main concept is underlined.

| **Narrative Story (The Tortoise and Hare)** | | |
| --- | --- | --- |
| MC | Version |  |
| 1 | Version 1 | The hare and the tortoise **raced**  兔仔 同埋 烏龜 **賽跑**  *tou3 zai2 tung4 maai4 wu1 gwai1* ***coi3 paau2*** |
|  | Version 2 | The hare and the tortoise **joined** a competition  兔仔 同埋 烏龜 **參加** 比賽  *tou3 zai2 tung4 maai4 wu1 gwai1* ***sam1 gaa1****bei2 coi3* |
| 2 | Version 1 | The hare (ran) **fast**er than the tortoise / so **fast**  兔仔 (跑得) **快**過 烏龜/ 好**快**  *tou3 zai2 (paau2 dak1)* ***faai3*** *gwo3 wu1 gwai1 / hou2* ***faai3*** |
|  | Version 2 | The hare **overtook** the tortoise  兔仔 **領先/拋離** 烏龜  *tou3 zai2* ***ling5 sin1/paau1 lei4*** *wu1 gwai1* |
| 3 | Version 1 | The hare thought (he) would **win** / The hare was **arrogant**  兔仔 覺得/諗住 **蠃**梗 / 兔仔 好 **驕傲**  *tou3 zai2 gok3 dak1/lam2 zyu6* ***jeng4****gang2 / tou3 zai2 hou2* ***giu1 ngou6*** |
|  | Version 2 | The hare thought the tortoise would **not arrive** shortly  兔仔 覺得/諗住 烏龜 有排 **未到**  *tou3 zai2 gok3 dak1/lam2 zyu6 wu1 gwai1 jau5 paai4* ***mei6 dou3*** |
|  | Version 3 | The hare thought the tortoise (ran) too **slow**  兔仔 覺得/諗住 烏龜 (跑得) 咁 **慢**  *tou3 zai2 gok3 dak1/lam2 zyu6 wu1 gwai1 (paau2* *dak1) gam3* ***maan6*** |
| 4 | - | The hare **slept**/**rested** for a while  兔仔 就 **瞓** 一陣  *tou3 zai2 zau6****fan3*** *jat1 zan6* |
| 5 | Version 1 | The tortoise **caught up**  烏龜 **追上/領先** 啦  *wu1 gwai1* ***zeoi1 soeng5 /ling5 sin1*** *laa1* |
|  | Version 2 | The tortoise **passed** the hare  烏龜 **超越** 兔仔  *wu1 gwai1* ***ciu1 jyut6*** *tou3 zai2* |
| 6 | - | (The tortoise) **ran** continually / (The tortoise) **ran** towards the destination  (烏龜) 不斷 **跑** / (烏龜) **跑**向 終點  *(wu1 gwai1) bat1 tyun5* ***paau2*** */* ***paau2*** *hoeng3 zung1 dim2* |
| 7 | - | The hare **woke up**  兔仔 **起身**/**瞓醒**  *tou3 zai2* ***hei2 san1****/****fan3 sing2*** |
| 8 | Version 1 | The tortoise **arrived** the destination  烏龜 **到達** 終點  *wu1 gwai1* ***dou3 daat6*** *zung1 dim2* |
|  | Version 2 | The tortoise **became** the champion  烏龜 **攞**到 冠軍  *wu1 gwai1* ***lo2*** *dou3 gun1 gwan1* |
|  | Version 3 | The tortoise **won**  烏龜 **蠃**/**勝出**  *wu1 gwai1* ***jeng4/sing3 ceot1*** |

Lexical items that are commonly accepted as alternatives in “Tortoise and Hare”

| MC | Essential | Alternatives |
| --- | --- | --- |
| 1，2，3，5，6，8 | 烏龜 | 小龜，小烏龜，阿龜，烏龜仔 |
| 1，2，3，4，7 | 兔仔 | 白兔，小兔，小兔子，兔子，阿兔 |
| 1 | 同埋 | 同，和 |
| 1 | 參加 [-比賽] | 進行，鬥 |
| 1 | [參加-] 比賽 | 跑步，跑步比賽，賽跑，賽跑比賽 |
| 2 | [兔仔-] 快 | 快啲，快好多，第一 |
| 3 | [覺得-] 蠃 | 跑第一，跑得快啲，快啲，快好多，蠃烏龜 |
| 3 | [烏龜-] 未到 | 追唔上，跟唔上 |
| 3，6 | [烏龜-] 跑 | 爬，行，走 |
| 4 | [兔仔-] 瞓 | 瞓覺，休息 |
| 5 | [烏龜-] 超越 | 趕過，爬過，行過 |
| 6 | [烏龜-] 不斷 | 繼續，一直，喺噉，一步一步，不停 |
| 8 | [烏龜-] 到達 | 先到，衝過，跑到 |
| 8 | [烏龜-] 攞 | 拎，得，成為 |
| 8 | [攞-] 冠軍 | 第一名，金牌 |
